# Translating AI research into reality: summary of the 2025 voice AI Symposium and Hackathon

**DOI:** 10.3389/fdgth.2026.1754426

**Published:** 2026-03-16

**Authors:** Samantha Salvi Cruz, Jamie Toghranegar, Bradley Malin, Tarun Mehra, Bob MacDonald, Marisha Speights, Camille Noufi, Yan Fossat, Guy Fagherrazi, Nicholas Cummins, Abir Elbeji, Alden Blatter, Alexander Gelbard, Arianna Arienzo, Sebastien Baur, Katie Wetstone, Julián Peller, Rhoda Au, Hugo Botha, Amir Lahav, Daria Hemmerling, Fabio Catania, James Anibal, Shumit Saha, Oita Coleman, Hortense Gallois, Sophia Avila Martinez, Nihar Mahapatra, Jaskanwal Deep Singh Sara, Aarush Mathur, Rupal Patel, Konrad Zieliński, Lampros Kourtis, Jordan Lerner-Ellis, Yan Cong, Hoan Ngo, Tanya Talkar, Greg Hale, Keith Comito, Satrajit Ghosh, Stephanie Watts, Steven Bedrick, Maria Powell, Jean-Christophe Bélisle-Pipon, Andrea Krussel, Ishaan Mahapatra, Ruth Bahr, Karim Hanna, Cynthia Kostelnik, Katie Dorsey, MyVan John, Kathleen Curp, Anaïs Rameau, The Bridge2AI-Voice Consortium, Yael Bensoussan

**Affiliations:** 1 Department of Otolaryngology – Head & Neck Surgery, Vanderbilt University Medical Center, Nashville, TN, United States; 2 Department of Otolaryngology – Head & Neck Surgery, University of South Florida, Tampa, FL, United States; 3 Department of Biomedical Informatics, Vanderbilt University Medical Center, Nashville, TN, United States; 4 Microsoft Health & Life Science, Boston, MA, United States; 5 Google, Mountain View, CA, United States; 6 Department of Communication Sciences & Disorders, Northwestern University, Evanston, IL, United States; 7 Amplifier Health, San Francisco, CA, United States; 8 Klick Applied Sciences, Toronto, ON, Canada; 9 Department of Precision Health, Luxembourg Institute of Health, Strassen, Luxembourg; 10 Institute of Psychiatry, Psychology & Neuroscience, King's College London, London, United Kingdom; 11 Faculty of Health Sciences, Simon Fraser University, Burnaby, BC, Canada; 12 VoiceMed, Rome, Italy; 13 Google Research, New York, NY, United States; 14 DrivenData, Washington, DC, United States; 15 Everything ALS Foundation, Seattle, WA, United States; 16 Department of Anatomy & Neurobiology, Chobanian & Avedisian School of Medicine, Boston University, Boston, MA, United States; 17 Department of Neurology, Mayo Clinic, Rochester, MN, United States; 18 SkyMedAI, Boston, MA, United States; 19 Department of Measurement and Electronics, AGH University of Krakow, Krakow, Poland; 20 McGovern Institute for Brain Research, Massachusetts Institute of Technology, Cambridge, MA, United States; 21 Department of Engineering Science, Institute of Biomedical Engineering, University of Oxford, Oxford, United Kingdom; 22 Department of Biomedical Data Science, School of Applied Computational Sciences, Meharry Medical College, Nashville, TN, United States; 23 Open Voice TrustMark Initiative, Apex, NC, United States; 24 Department of Electrical & Computer Engineering, Michigan State University, East Lansing, MI, United States; 25 Department of Cardiovascular Medicine, Mayo Clinic, Rochester, MN, United States; 26 Department of Communication Sciences and Disorders, Northeastern University, Boston, MA, United States; 27 Uhura Bionics, Mazowieckie, Poland; 28 ADDF SpeechDx Program, Boston, MA, United States; 29 Department of Pathology and Laboratory Medicine, Mount Sinai Hospital, Toronto, ON, Canada; 30 Department of Laboratory Medicine and Pathobiology, University of Toronto, Toronto, ON, Canada; 31 School of Languages and Cultures, Purdue University, West Lafayette, IN, United States; 32 Department of Computer Science and Engineering, Florida Southern College, Lakeland, FL, United States; 33 Linus Health, Cambridge, MA, United States; 34 Walt Disney Park and Resorts, Orlando, FL, United States; 35 Lifespan Extension Advocacy Foundation, Seaford, NY, United States; 36 Medical Informatics & Clinical Epidemiology, Oregon Health & Science University, Portland, OR, United States; 37 Department of Health Sciences, Simon Fraser University, Burnaby, BC, Canada; 38 Health Information and Data Science, Washington University in St. Louis, St. Louis, MO, United States; 39 Haslett High School, Haslett, MI, United States; 40 Department of Communication Sciences and Disorders, University of South Florida, Tampa, FL, United States; 41 Office of Research, University of South Florida, Tampa, FL, United States; 42 Office of Development and Alumni Relations, University of South Florida, Tampa, FL, United States; 43 Office of Communication and Marketing, University of South Florida, Tampa, FL, United States; 44 Department of Otolaryngology – Head & Neck Surgery, Weill Cornell Medicine, New York, NY, United States

**Keywords:** artificial intelligence, clinical translation, multimodal data, vocal biomarkers, voice AI

## Abstract

The 2025 Voice AI Symposium represented a transition from conceptual research to clinical implementation in vocal biomarker science. Hosted by the NIH-funded Bridge2AI-Voice consortium, the meeting convened global experts to address the methodological, ethical, and translational challenges of integrating voice-based artificial intelligence (AI) into healthcare. This mini-review synthesizes symposium insights across six domains: multimodal integration, FAIR (Findable, Accessible, Interoperable, Reusable) and CARE (Collective Benefit, Authority to Control, Responsibility, Ethics) data governance, clinical translation, interdisciplinary training, and cross-sector innovation. Research presented demonstrated voice as a latent, multimodal biomarker reflecting neurological, cardiopulmonary, and psychological states, while discussions emphasized ethical data practices and human-centered design. The implementation-focused panels underscored the importance of workflow alignment and usability for adoption in real-world care. Collectively, the symposium reflects a field advancing toward translational readiness and ethical accountability, positioning voice AI as a scalable, inclusive tool for next-generation healthcare.

## Introduction

The Voice AI Symposium, hosted by the Bridge2AI-Voice consortium, is an annual event to foster collaboration, engagement, and innovation around the use of vocal biomarkers in healthcare. While previous years emphasized foundational research, the 2025 Symposium reflected a pivotal shift in the field towards implementation. International researchers, thought leaders, startup owners, clinicians, and regulators gathered in Tampa, Florida to collaborate and discuss the practical validation, deployment and integration of vocal biomarkers into healthcare delivery systems. Throughout the symposium, scientific advancements were discussed alongside ethical challenges and infrastructural requirements for clinical translation. The following conference proceedings serve as an overview and thematic discussion of the 2025 event.

## Background

Voice is increasingly recognized as a non-invasive, data-rich biomarker of health, capable of capturing a wide range of physiological and psychological states (1). As a multidimensional signal tied to respiratory, cognitive, neurological, and emotional function, voice provides clinical insight into both primary voice disorders as well as voice-affecting conditions (1–3). Artificial intelligence (AI) and machine learning models are increasingly used to detect subtle acoustic features in voice recordings that are often undetectable to the human ear, but may signal early signs of disease (4, 5). AI models trained on vocal features have potential to operate within tools to assist in disease identification, classification, and dynamic monitoring of health states. The rise of wearable technologies and telemedicine has made it increasingly feasible to collect voice data passively and at scale (1, 3, 6). Despite these advancements, however, complex challenges remain, including the need for ethically-sourced, standardized, and AI-ready datasets, as well as best practices for data privacy, consent, and mitigating algorithmic bias (4).

The National Institutes of Health (NIH)-funded Bridge2AI-Voice program is seeking to address these challenges by building a large, ethically sourced, multimodal voice database linked to clinical data. Using multi-disorder protocols built by interdisciplinary teams of experts, Bridge2AI-Voice data is collected using standardized recording protocols and is then clinically validated by physicians and healthcare providers. The robust Bridge2AI-Voice dataset supports the development of predictive models across five clinical domains: voice disorders, respiratory conditions, mood disorders, neurological diseases, and pediatric speech and language disorders. This coordinated, multi-site effort is also producing the associated tools and training resources for reproducible, ethical, and scalable clinically meaningful vocal biomarker research.

To further scientific discovery and collaboration among researchers in this field, Bridge2AI-Voice hosts an annual symposium that brings together experts from academia, healthcare, industry, bioethics, and patient advocacy groups. The following represent conference proceedings of the 2025 Voice AI Symposium. This paper is organized around the thematic insights and scientific discussions presented at the 2025 Bridge2AI Voice Symposium, emphasizing methodological innovations, ethical considerations, and strategies for integrating vocal biomarkers into clinical practice.

### Format

The 2025 Voice AI Symposium presented a comprehensive program reflecting both scientific depth and translational relevance. The meeting featured two keynote speeches: Dr. Bradley Malin [*“How Synthetic Data Can (and Cannot) Help Privacy in Voice Datasets”*] and Microsoft's Tarun Mehra (*“Voice AI and the Future of Healthcare”*). In addition to these plenary sessions, the symposium included interactive workshops, a startup pitch competition, poster presentations, a tech fair, and structured networking opportunities. Four podium and panel sessions occurred with the following themes: “*Emerging Research Methods and Technologies”; “Ethical Translation and Clinical Implementation of Voice AI”; “Implementation of Acoustic Biomarkers of Airway and Cardiorespiratory Disorders”;* and “*Advancements in Voice Biomarkers of Neurological Diseases”.* Abstracts were selected through a competitive call-for-science process, and all submissions underwent peer review by a scientific committee comprising internal and external experts in the field, thereby ensuring the quality, rigor, and diversity of the program.

### Methods

All symposium presentations were recorded in full. Audio files were uploaded to Whisper v3 (OpenAI, 2025), which generated transcripts for each session. These transcripts were then processed using ChatGPT (v4) to identify recurring scientific insights, conceptual patterns, and cross-disciplinary themes. The resulting thematic clusters informed the structure of this paper.

### Results

The following results are organized around 6 emergent themes identified through transcript analysis of all recorded events. These themes included the multimodality of voice data, Ambient AI Scribes and Ethical Implications of Continuous Voice Capture, FAIR (Findable, Accessible, Interoperable, Reusable) and CARE (Collective Benefit, Authority to Control, Responsibility, Ethics) for voice AI, translational readiness of vocal biomarkers, the need for interdisciplinary training, and innovation through cross-sector engagement. The following is a summation and discussion of the most pressing and high-impact scientific themes that emerged at this year's event.

### An exploration of voice and multimodality

Research presented at the 2025 event reflected an emerging theme: the concept of voice as a latent, multimodal biomarker capable of providing a holistic lens on health beyond discrete symptom tracking. While previous studies have explored vocal features in neurological, cardiopulmonary, and psychiatric contexts, this year's presentations added empirical depth and interdisciplinary range, presenting voice as a complex, integrative signal with diagnostic, prognostic, and monitoring potential (1, 7). Rather than pointing to isolated conditions, vocal patterns increasingly reflect the interaction of physiological, neurological, and psychological systems. This evolving perspective is shifting the field from narrow, disease-specific modeling to broader, systems-level approaches. Vocal markers, such as articulation rate, prosody, timing, and lexical variation, are being investigated as early signals of general physiological decline (4). Researchers are now turning to probabilistic, multi-output models that can link these features to composite health states, which mirrors the complexity of comorbid, real-world populations. Dr. Lampros Kourtis illustrated this approach with findings from the Framingham Heart Study (8), which paired over 4,000 voice recordings with MRI-derived brain volume data. Vocal markers like jitter, articulation rate, and lexical diversity were significantly associated with structural changes in memory-related brain regions (8). In cardiopulmonary research, Dr. Jas Sara's team developed models, leveraging both HuBERT and traditional signal-processing approaches, that detect subtle changes in breath support, phonatory control, and speech timing. These tools flag early signs of decompensation in heart failure patients and outperform standard clinical indices, offering clinicians a more proactive and cost-effective way to manage chronic care. Together, these studies reflect a growing consensus: voice is a sensitive, often pre-symptomatic, marker of physiological change.

### Ambient AI scribes and ethical implications of continuous voice capture

Recent advances in voice-based AI have highlighted ambient AI scribe systems as a promising interface between vocal biomarker research and clinical deployment. In his keynote, Tarun Mehra emphasized that ambient scribe technologies, originally designed to reduce documentation burden, are rapidly evolving into platforms capable of passively capturing clinically meaningful vocal signals during routine care encounters. This shift reframes voice not only as an active diagnostic input but as a continuously acquired signal embedded within everyday clinical workflows. However, symposium discussions highlighted that this paradigm introduces distinct ethical and governance challenges that extend beyond traditional voice AI use cases. Unlike task-based recordings, ambient scribe systems collect speech continuously, raising questions around consent granularity, secondary data use, speaker identifiability, and power asymmetries between patients, clinicians, and health systems. Ethical panels and Dr. Bradley Malin's keynote emphasized that safeguards such as synthetic data, de-identification, and auditability are necessary but insufficient. They further underscored the need for participatory governance models and transparency around how ambient voice data are repurposed for model development. Importantly, speakers cautioned that ambient AI scribes risks amplifying existing inequities if deployed without attention to linguistic diversity, sociocultural context, and algorithmic bias, particularly given the variability of speech across dialects, health states, and care settings. Collectively, these discussions highlighted that ambient AI scribes requires coordinated attention to technical performance, ethical governance, and real-world evaluation to support clinical use.

### FAIR and CARE in voice AI

Researchers and scientists are facing increasing pressure to understand what are best practices for ethical voice data governance. At this year's symposium, speakers addressed these concerns and emphasized that advances in voice AI must be guided by governance frameworks grounded in both FAIR and CARE principles (9, 10). Dr. Bradley Malin's keynote on synthetic data highlighted both its promise for protecting voice privacy and its limitations, underscoring the need for nuanced consent and governance protocols given the uniquely personal nature of voice. During a panel discussion, it was discussed that 38 publicly available voice repositories have significant gaps in metadata, demographic representation, and transparency, illustrating risks to both FAIR and CARE objectives. Speakers stressed that governance must remain iterative and participatory, with human oversight and interpretability central to ethical implementation. As Dr. Rhoda Au noted, equity in voice AI extends beyond datasets to the diversity of teams developing these technologies. Embedding FAIR and CARE principles throughout design, governance, and practice is essential for building trust and ensuring responsible scale-up in healthcare applications (1).

### Translational readiness: real-world clinical integration

**Figure 1:**
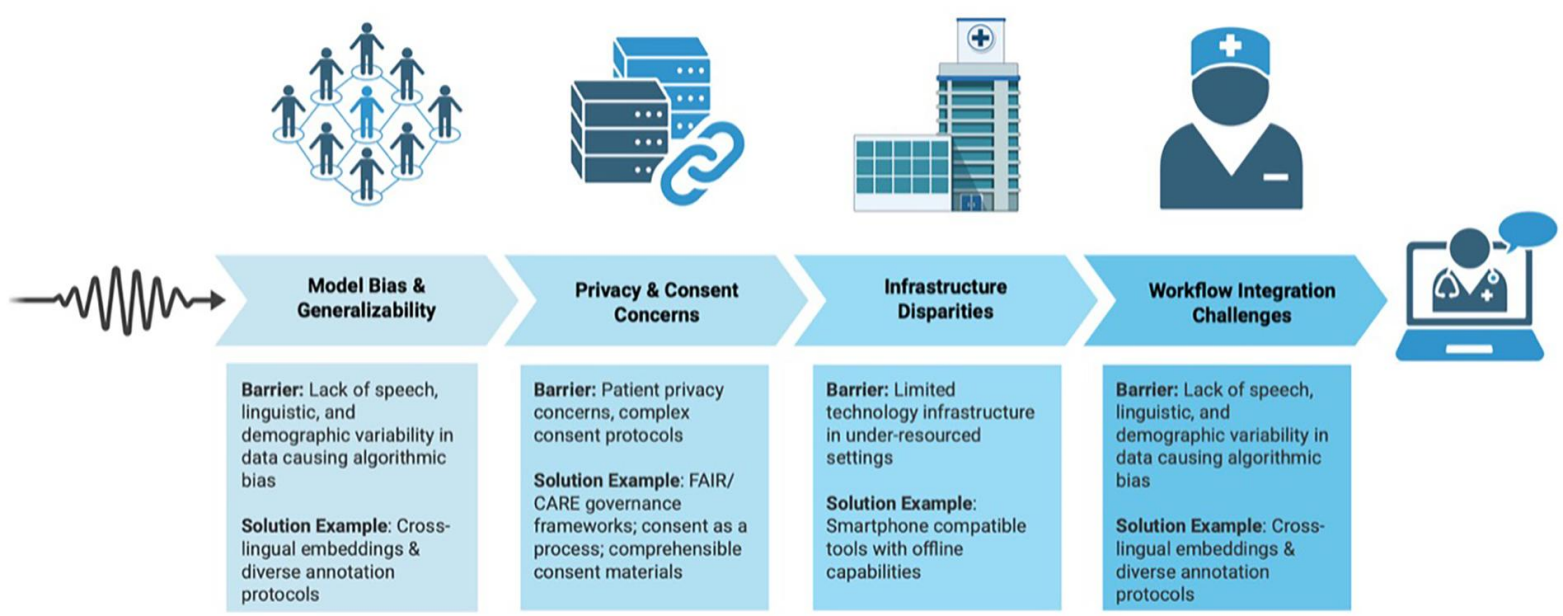
Vocal biomarker implementation barriers & solutions in clinical care.

As the vocal biomarker field advances and evolves, there is increasing interest in translating findings into real-world clinical integration. Dr. Hugo Botha offered a physician's perspective on this challenge. His team has piloted voice-based decision support tools in post-operative care units, using speech analysis to monitor recovery and flag early signs of complications. These tools have the potential to streamline documentation and enhance care team coordination. Still, Botha emphasized that successful deployment depends on seamless integration. Voice tools must align with existing clinical workflows, electronic health record systems, and the everyday communication patterns of providers; otherwise, they risk becoming burdensome rather than beneficial. Expert panelists discussed barriers to real-world implementation, including time-constrained workflows, infrastructure disparities, and valid concerns about patient privacy (see Figure 1). Many advocated for low-burden, device-agnostic tools that operate within existing clinical ecosystems, especially in under-resourced settings.

Other important considerations for effective implementation include the need to mitigate algorithmic bias. An educational workshop provided a deep-dive discussion on incorporating linguistic and speaker variability into datasets to ensure robust translation of voice AI technologies into real-world clinical and social contexts. Dr. Satrajit Ghosh's team offered technical strategies to support this aim, including cross-lingual embeddings and culturally attuned annotation protocols. They emphasized that achieving translational success requires technical accuracy alongside design choices that foster usability, trust, and alignment with care delivery practices.

### The need for interdisciplinary training for voice AI researchers

Building AI models with voice data for clinical applications introduces challenges unfamiliar to many computer scientists, requiring integration of biomedical, clinical, and ethical expertise. The 2025 Voice AI Symposium offered interactive educational workshops on Bridge2AI-Voice dataset and resources, as well as a full day Hackathon event with a non-programmer educational track to provide in-depth training on how to ethically and effectively build models with voice data for healthcare applications. Workshops included a live videostroboscopy-demo, illustrating the physiological basis of vocal signals to attendees, as well as a demonstration of how to use the Bridge2AI-Voice data and tools, and in-depth discussion of data standards. Collectively, these initiatives highlighted the imperative to cultivate hybrid professionals with both technical and clinical competencies.

### Translational innovation and cross-sector engagement

A key component of the Voice AI Symposium was also engagement with industry and startup sectors who are translating and developing voice AI solutions into healthcare tools. Use cases presented by participating teams in the pitch competition spanned depression risk screening, congestive heart failure monitoring, assistive communication technologies, and occupational health applications, reflecting the breadth of proposed clinical and non-clinical deployments for voice-based models. Importantly, the evaluation criteria emphasized usability, workflow integration, and readiness for prospective clinical validation, underscoring that translational success depends on alignment with real-world care environments rather than model performance alone.

## Discussion

The 2025 Bridge2AI Voice Symposium reflected a field advancing from early proof-of-concept studies toward the practical challenges of clinical translation. Presentations demonstrated how acoustic features can reflect interactions among neurological, cardiopulmonary, and psychological systems, pointing to a broader systems-level framing of health (2, 8). This shift marks an important maturation: rather than treating voice as a disease-specific signal, researchers are increasingly positioning it as an integrative marker of overall physiological state. The discussions suggested that this conceptual transition is shaping the future of the field more than any single technical advance. Methodologically, researchers are continuing to refine multimodal integration strategies and develop analytic approaches that can handle both population diversity and individual variability over time (11,12,13).

Workforce development is also a parallel imperative. It is imperative that we build a pipeline of researchers who are trained to work across computer science, biomedicine, and ethics will be essential for sustaining progress. Finally, international governance frameworks specific to voice data are still being developed to ensure that datasets and models are built responsibly and equitably (9, 10). Together, these directions point toward a field that is increasingly translational, but also reflective about the conditions under which voice AI can become a reliable and inclusive part of healthcare practice.

## Conclusion

Voice AI is entering its translational phase, moving from possibility to clinical practice. This mini-review captures a pivotal moment in that journey: where breakthroughs in science, ethics, and tooling converged toward actionable pathways. However, continued progress will require collective accountability, with community values shaping consent protocols, governance frameworks, and the practical decisions around when, where, and how voice technologies are deployed.

Voice carries diagnostic power, but also memory, identity, and emotional resonance. As we continue to build, test, and deploy these technologies, we must remain attuned to both. Through sustained collaboration and ethical vigilance, voice AI can become a foundation for care that is intelligent, inclusive, and genuinely responsive.

## The Bridge2AI-voice consortium

Yael E. Bensoussan, University of South Florida, Tampa, FL, USA; Olivier Elemento, Weill Cornell Medicine, New York, NY, USA; Jean-Christophe Bélisle-Pipon, Simon Fraser University, Burnaby, BC, Canada; David Dorr, Oregon Health & Science University, Portland, OR, USA; Satrajit Ghosh, Massachusetts Institute of Technology, Boston, MA, USA; Alistair Johnson, Microsoft, Toronto, ON, Canada; Phillip Payne, Washington University in St. Louis, St. Louis, MO, USA; Maria E. Powell, Vanderbilt University Medical Center, Nashville, TN, USA; Anaïs Rameau, Weill Cornell Medicine, New York, NY, USA; Vardit Ravitsky, The Hastings Center, Garrison, NY, USA; Alexandros Sigaras, Weill Cornell Medicine, New York, NY, USA; Jordan Lerner-Ellis, University of Toronto, Toronto, ON, Canada; Shaheen Awan, University of Central Florida, Orlando, FL, USA; Ruth Bahr, University of South Florida, Tampa, FL, USA; Frank Rudzicz, Dalhousie University, Toronto, ON, Canada; Jennifer Siu, Hospital for Sick Children, Toronto, ON, Canada; Stephanie Watts, University of South Florida, Tampa, FL, USA; Yassmeen Abdel-Aty, University of South Florida, Tampa, FL, USA; Steven Bedrick, Oregon Health & Science University, Portland, OR, USA; Alexander Bernier, McGill University, Montreal, QC, Canada; Micah Boyer, University of South Florida, Tampa, FL, USA; Moroni Berrios, University of South Florida, Tampa, FL, USA; John Brown, University of South Florida, Tampa, FL, USA; Kimberly Vinson, Vanderbilt University Medical Center, Nashville, TN, USA; Kenneth Fletcher, Vanderbilt University Medical Center, Nashville, TN, USA; Hortense Gallois, Simon Fraser University, Burnaby, BC, Canada; C. Gaelyn Garrett, Vanderbilt University Medical Center, Nashville, TN, USA; Alexander Gelbard, Vanderbilt University Medical Center, Nashville, TN, USA; Amer Ghavanini, Trillium Health Partners and University of Toronto, Toronto, ON, Canada; Karim Hanna, University of South Florida, Tampa, FL, USA; William Hersh, Oregon Health & Science University, Portland, OR, USA; Jennifer Jain, University of South Florida, Tampa, FL, USA; Kenji Kobayashi, Vanderbilt University Medical Center, Nashville, TN, USA; Andrea Krussel, Washington University in St. Louis, St. Louis, MO, USA; Sarah Rohde, Vanderbilt University Medical Center, Nashville, TN, USA; Jillian Rossi, University of South Florida, Tampa, FL, USA; Laurie Russell, SickKids, Toronto, ON, Canada; Samantha Salvi Cruz, Vanderbilt University Medical Center, Nashville, TN, USA; Shrramana Ganesh Sudhakar, University of South Florida, Tampa, FL, USA; Megan Urbano, University of South Florida, Tampa, FL, USA; Theresa Zesiewicz, University of South Florida, Tampa, FL, USA; Jamie Toghranegar, University of South Florida, Tampa, FL, USA; Cynthia Kostelnik, University of South Florida, Tampa, FL, USA; James Anibal, University of Oxford, Oxford, UK; Kirollos Armosh, University of South Florida, Tampa, FL, USA; Helena Beltran, University of South Florida, Tampa, FL, USA; Isaac Bevers, Massachusetts Institute of Technology, Cambridge, MA, USA; Rahul Brito, Massachusetts Institute of Technology, Cambridge, MA, USA; Mahmoud Elmahdy, University of South Florida, Tampa, FL, USA; Evan Ng, SickKids, Toronto, ON, Canada; Kaley Jenney, Massachusetts Institute of Technology, Cambridge, MA, USA; Megha Kalia, University of South Florida, Tampa, FL, USA; Alisa Krause, University of South Florida, Tampa, FL, USA; Genelle Leo, University of South Florida, Tampa, FL, USA; Chloe Loewith, Simon Fraser University, Burnaby, BC, Canada; Siyu Miao, SickKids, Toronto, ON, Canada; Tempestt Neal, University of South Florida, Tampa, FL, USA; Claire Premi-Bortolotto, Simon Fraser University, Burnaby, BC, Canada; John Ramos, Weill Cornell Medicine, New York, NY, USA; John Stark, University of South Florida, Tampa, FL, USA; Venkata Swarna Mukhi, Oregon Health & Science University, Portland, OR, USA; Julie Tu, Mount Sinai Hospital, Toronto, ON, Canada; Claire Wilson, Simon Fraser University, Burnaby, BC, Canada; Madeleine Zanin, Mount Sinai Hospital, Toronto, ON, Canada; Mohamed Ebraheem, University of South Florida, Tampa, FL, USA; Emily Evangelista, University of South Florida, Tampa, FL, USA; Anna Goldenberg, SickKids, Toronto, ON, Canada; Ayush Kalia, University of South Florida, Tampa, FL, USA; Vrishni Maharaj, University of South Florida, Tampa, FL, USA; Ana Sophia Avila Martinez, University of South Florida, Tampa, FL, USA; Elijah Moothedan, Florida Atlantic University, Boca Raton, FL, USA; JM Warith Rahman, University of South Florida, Tampa, FL, USA; Jordan Wilke, Massachusetts Institute of Technology, Cambridge, MA, USA.
